# Maternal antenatal depression is associated with metabolic alterations that predict birth outcomes, neurodevelopment and mental health of the child

**DOI:** 10.1192/j.eurpsy.2025.346

**Published:** 2025-08-26

**Authors:** P. Girchenko

**Affiliations:** 1 Clinical Medicine Research Unit, MRC Oulu, University of Oulu, Oulu; 2 Deoartment of Psychology, University of Helsinki, Helsinki, Finland

## Abstract

**Introduction:**

Evidence regarding metabolic alterations associated with maternal antenatal depression (AD) is limited, and their role as potential biomarkers improving the prediction of AD and adverse child birth, neurodevelopmental, and mental health outcomes remains unexplored.

**Objectives:**

To identify metabolic measures associated with AD.

To test whether the metabolic measures associated with AD increased the amount of variance explained in AD over its risk factors.

To test whether the identified metabolic measures increased the amount of variance explained in child gestational age and weight at birth, developmental milestones at ages 2.3-5.7 years and any mental or behavioral disorder by the ages of 13.1-16.8 years over AD, sex, and age.

To replicate the findings in an independent cohort.

**Methods:**

In a cohort of 331 mother-child dyads, we applied elastic net regression to study associations between AD (history of medical register diagnoses and/or Center of Epidemiological Studies Depression Scale score during pregnancy≥20) and 95 metabolic measures analyzed three times during pregnancy. Child birth and mental health outcomes were extracted from national registers and child neurodevelopmental outcomes were mother-reported.

**Results:**

Elastic net regression identified 15 metabolic measures that collectively explained 25% (p<0.0001) of variance in AD, including amino and fatty acids, glucose, inflammation, and lipids. These metabolic measures increased the variance explained in AD over its risk factors (32.3%,p<0.0001 vs. 12.6%,p=0.004), and in child gestational age (9.0%,p<0.0001 vs. 0.7%, p=0.34), birth weight(9.0%,p=0.03 vs. 0.7%, p=0.33), developmental milestones at the age of 2.3-5.7 years(21.0%,p=0.002 vs. 11.6%,p<0.001) and any mental or behavioral disorder by the age of 13.1-16.8 years(25.2%,p=0.03 vs. 5.0%,p=0.11) over AD, child sex and age. These findings replicated in the independent cohort.

**Conclusions:**

AD is associated with alterations in 15 metabolic measures, which collectively improve the prediction of AD over its risk factors, and birth, neurodevelopmental and mental health outcomes of the child over AD. These metabolic measures may become biomarkers identifying at-risk mothers and children for personalized interventions.

**Disclosure of Interest:**

None Declared

